# Balancing constraints and requirements in an application process

**DOI:** 10.23889/ijpds.v9i5.2608

**Published:** 2024-09-18

**Authors:** Stela McLachlan, Katharine Evans, Rebecca Whitehorn, Emma Turner, Jacqueline Oakley, Andy Boyd, Robin Flaig

**Affiliations:** 1 University of Edinburgh; 2 University of Bristol

## Objectives

Developing a rigorous application process for linked data within the UK Longitudinal Linkage Collaboration’s Trusted Research Environment (TRE) included balancing the requirements of >20 longitudinal studies and several data providers with the need to provision data to researchers in a safe, ethical and efficient manner.

## Approach

UK LLC was commissioned as a resource to enable the investigation of COVID-19 questions: a secure setting where longitudinal population studies (LPS) can be linked efficiently to regularly refreshed NHS electronic health records, and then accessed by researchers. UK LLC received funding to continue as a resource for any research in public good and to link to the administrative records (education, occupation, tax and benefits). To accommodate the constraints imposed by a new set of data controllers and to account for wide variety of proposals expected, we expanded our data access process.

## Results

UK LLC has a three-stage application and review process. The addition of administrative data meant that there is additional review for those applications requesting that data (Stage 4) and need to align processes with the UK Statistics Authority. There is also need to review complexity of the data requests and ethical implications of any application to access the data. We have developed an ethics decision tree to help guide the decision making around ethics requirements.

## Conclusion

Our process is evolving as we are trying to meet the challenge of balancing the needs of >20 studies, stakeholders, applicants, and participant/public contributors, to create a process that can handle complex needs and requirements.

